# Robust Performance of Potentially Functional SNPs in Machine Learning Models for the Prediction of Atorvastatin-Induced Myalgia

**DOI:** 10.3389/fphar.2021.605764

**Published:** 2021-04-22

**Authors:** Brandon N. S. Ooi, Raechell, Ariel F. Ying, Yong Zher Koh, Yu Jin, Sherman W. L. Yee, Justin H. S. Lee, Samuel S. Chong, Jack W. C. Tan, Jianjun Liu, Caroline G. Lee, Chester L. Drum

**Affiliations:** ^1^ Department of Biochemistry, Yong Loo Lin School of Medicine, National University of Singapore, Dundee, Singapore; ^2^ Duke-NUS Graduate School, Singapore, Singapore; ^3^ Division of Cellular and Molecular Research, Humphrey Oei Institute of Cancer Research, National Cancer Centre Singapore, Singapore, Singapore; ^4^ Department of Medicine, Yong Loo Lin School of Medicine, Cardiovascular Research Institute, National University of Singapore, Singapore, Singapore; ^5^ NovogeneAIT Genomics Singapore, Singapore, Singapore; ^6^ Department of Pediatrics, Yong Loo Lin School of Medicine, National University of Singapore, Singapore, Singapore; ^7^ Department of Cardiology, National Heart Centre Singapore, Singapore, Singapore; ^8^ Genome Institute of Singapore, Singapore, Singapore; ^9^ NUS Graduate School for Integrative Sciences and Engineering, National University of Singapore, Singapore, Singapore; ^10^ Translational Laboratory in Genetic Medicine, Singapore, Singapore

**Keywords:** statin, myalgia, whole genome sequencing, machine learning, pharmacogenomics

## Abstract

Statins can cause muscle symptoms resulting in poor adherence to therapy and increased cardiovascular risk. We hypothesize that combinations of potentially functional SNPs (pfSNPs), rather than individual SNPs, better predict myalgia in patients on atorvastatin. This study assesses the value of potentially functional single nucleotide polymorphisms (pfSNPs) and employs six machine learning algorithms to identify the combination of SNPs that best predict myalgia.

**Methods:** Whole genome sequencing of 183 Chinese, Malay and Indian patients from Singapore was conducted to identify genetic variants associated with atorvastatin induced myalgia. To adjust for confounding factors, demographic and clinical characteristics were also examined for their association with myalgia. The top factor, sex, was then used as a covariate in the whole genome association analyses. Variants that were highly associated with myalgia from this and previous studies were extracted, assessed for potential functionality (pfSNPs) and incorporated into six machine learning models. Predictive performance of a combination of different models and inputs were compared using the average cross validation area under ROC curve (AUC). The minimum combination of SNPs to achieve maximum sensitivity and specificity as determined by AUC, that predict atorvastatin-induced myalgia in most, if not all the six machine learning models was determined.

**Results:** Through whole genome association analyses using sex as a covariate, a larger proportion of pfSNPs compared to non-pf SNPs were found to be highly associated with myalgia. Although none of the individual SNPs achieved genome wide significance in univariate analyses, machine learning models identified a combination of 15 SNPs that predict myalgia with good predictive performance (AUC >0.9). SNPs within genes identified in this study significantly outperformed SNPs within genes previously reported to be associated with myalgia. pfSNPs were found to be more robust in predicting myalgia, outperforming non-pf SNPs in the majority of machine learning models tested.

**Conclusion:** Combinations of pfSNPs that were consistently identified by different machine learning models to have high predictive performance have good potential to be clinically useful for predicting atorvastatin-induced myalgia once validated against an independent cohort of patients.

## Introduction

Cardiovascular disease is a leading cause of death worldwide (World Health Organization – Cardiovascular Disease, 2020). High blood cholesterol levels increase the risk of cardiovascular disease, making lipid-lowering medications such as statins important for the therapeutic management of this risk factor (SEARCH Collaborative Group et al., 2010; Cholesterol Treatment Trialists et al., 2012; Silverman et al., 2016). Statins, or 3-hydroxy-3-methylglutaryl CoA reductase inhibitors, are generally well tolerated. However up to 25% of individuals have reported some degree of statin-associated muscle symptoms (SAMS) (Bruckert et al., 2005; Cohen et al., 2012). These side effects range from myalgia (with or without elevations in serum creatine kinase) to severe rhabdomyolysis (Alfirevic et al., 2014). Although severe forms of muscle toxicity such as myopathy and rhabdomyolysis are rare, the most common event leading to discontinuation of statins are muscle symptoms, in particular those without significant elevation in creatine kinase (McGinnis et al., 2007; Wei et al., 2013; Stroes et al., 2015). As treatment of hypercholesterolaemia is life-long, poor adherence to prescribed statin therapy increases the risk of cardiovascular events (Chowdhury et al., 2013; Saxon and Eckel, 2016). It is therefore important to be able to identify patients with muscle symptoms of pharmacological origin so that they can receive appropriate management. These patients could also receive alternative non-statin therapies such as the more expensive PSCK9 inhibitors or ezetimibe (Bakar et al., 2018).

Previous pharmacogenomic studies have reported genetic variations that are associated with SAMS, most notably the rs4149056 polymorphism in the *SLCO1B1* gene. (Link et al., 2008; Wilke et al., 2012). This polymorphism has been included in CPIC guidelines for simvastatin therapy. While the pharmacokinetic basis of rs4149056 and simvastatin-induced myopathy has been established in several clinical studies (Pasanen et al., 2006; Voora et al., 2009; Carr et al., 2013), it is unclear whether this variant is also associated with SAMS in patients on lower doses of statin, milder myalgia or from different populations (Donnelly et al., 2011.; Hubacek et al., 2015; Sai et al., 2016; Zhong et al., 2018). For instance, Donnelly et al. (2011) reported an association of *SLCO1B1* variants with mild myalgia in patients receiving high doses of statin, but Huback et al. (2015) reported that *SLCO1B1* polymorphisms were not associated with risk of myalgia in a Czech population. Furthermore, this association is strongest for simvastatin, and there are conflicting reports for atorvastatin treatment which is the most widely prescribed high-potency statin (Voora et al., 2009; Carr et al., 2013; Brunham et al., 2018). Atorvastatin, simvastatin and other statins differ in the ring that is attached to their active moieties as well as in the form that they are administered in (Turner and Pirmohamed, 2019; Ward et al., 2019). These statins therefore have different pharmacokinetic characteristics and involve different genes and SNPs for their metabolism and transport.

In addition to SNPs in the *SLCO1B1* gene, SNPs in several other pathways including statin metabolism [e.g., cytochrome P450 (CYP) genes (Frudakis et al., 2007; Shek et al., 2017) and glycine amidinotransferase (GATM) (Mangravite et al., 2013)], statin transport [e.g., ATP binding cassette (ABC) transporters (Zhang et al., 2019)], and immune response (e.g human leukocyte antigen (HLA) (Sai et al., 2016) and leukocyte immunoglobulin-like receptor (LILR) (Siddiqui et al., 2017)] have also been implicated in SAMS (reviewed in Ward et al., 2019; Turner and Pirmohamed, 2019). For some of these SNPs, further studies have shown that the associations do not replicate (e.g., the *GATM* variant) (Floyd et al., 2014; Luzum et al., 2015). Clinical factors such as age, sex, ethnicity, daily dose, body mass index, drug-drug interactions, comorbidities, duration of statin use and use of concomitant medications have also been implicated with SAMS (SEARCH Collaborative Group et al., 2010; Cohen et al., 2012; Tournadre, 2020), although the association of these covariates again varies with each study.

Hence, this study aims to examine the role of genetic and clinical factors for predicting atorvastatin-induced myalgia in the Singapore population, which comprises mainly of individuals of Chinese, Malay and Indian descent. Genetic polymorphisms associated with myalgia were obtained by whole genome sequencing (WGS). Unlike exome or targeted sequencing technologies previously used in the discovery of statin associated myopathy variants (Ruano et al., 2007; Ruano et al., 2011; Bakar et al., 2018; Floyd et al., 2019), WGS allows for the detection of polymorphisms in both coding and non-coding regions. Furthermore, our group has found that non-coding regions contain a larger proportion of potentially functional SNPs compared to coding regions (Bachtiar et al., 2019a), which makes WGS a more suitable platform compared to other technologies. Floyd et al. (2019) reported that there was no evidence linking rare coding variants to adverse statin reactions, and given our small sample size, we have decided to focus on common variants in this study.

The potentially functional SNPs (Wang et al., 2011) uncovered from this study, as well as from other known genes in the atorvastatin pathway, were used for predicting myalgia using a variety of machine learning approaches. Machine learning has previously been used to predict drug response or dosage in fields such as cancer, psychiatry and cardiovascular disease (Liu et al., 2015; Huang et al., 2018; Athreya et al., 2019). To our knowledge, it has not been applied to predict the risk of statin-induced myalgia based on pharmacogenomic data. Insights gained from this study can therefore help to reveal important clinical and genetic risk factors that are predictive of atorvastatin-induced myalgia, as well as demonstrate the utility of machine learning approaches in pharmacogenomics.

## Materials and Methods

### Study Cohort

This study examined 183 subjects on atorvastatin therapy from the Surveillance and Pharmacogenomics Initiative for Adverse Drug Reactions (SAPhIRE) project. Written informed consent was obtained from all participants and the study protocol was approved by the National Healthcare Group Domain Specific Review Board (NHG DSRB). For patients who reported muscle pain, severity of symptoms was scored based on two criteria, regional distribution pattern and temporal pattern. The scoring for regional distribution is as follows - “non-specific, intermittent” was given a score of 1, “symmetric hip flexors/thigh aches” was given a score of 3 while “symmetric calf aches” and “symmetric upper proximal aches” were given a score of 2. For temporal pattern, “onset < 4 weeks” was given a score of 3, “4–12 weeks” was given a score of 2 and “>12 weeks” was given a score of 1. The scores for the two patterns were added with scores ranging from 0 (no muscle pain) to 6, and patients who responded with a score of 0–2 were defined as the statin tolerant group while those with a score of 4–6 were defined as the myalgia group. All 30 patients in the myalgia group were selected for further analysis, while 153 out of 946 patients were randomly selected from the atorvastatin tolerant group to form the controls. From this study cohort, 48% were self-reported Chinese, 31% Indian and 21% Malay, and patients had a mean age of 57.4 (95% CI: 55.9–59.0) years, although one patient did not have age data. Patients were treated with atorvastatin for 10–5,046 days with a daily dose ranging from 5 to 80 mg. Demographics (including age, sex, height and weight), comorbidities and medications of all patients were recorded. Each patient provided a venous blood sample which was transferred into EDTA tubes and stored at −80°C for genetic analyses.

### Whole Genome Sequencing

Genomic DNA was extracted and purified from whole blood using the Omega Bio-Tek E. Z.N.A. Blood DNA mini kit (Norcross, GA, United States). DNA concentration was measured using Qubit® DNA Assay Kit in Qubit® 2.0 Flurometer (Life Technologies, CA, United States). Fragment distribution of DNA library was measured using the DNA Nano 6000 Assay Kit of Agilent Bioanalyzer 2100 system (Agilent Technologies, CA, United States). A total amount of 1.0 μg DNA per sample was used as input material for the DNA sample preparations. Sequencing libraries were generated using NEBNext® DNA Library Prep Kit following manufacturer’s recommendations and indices were added to each sample. The genomic DNA is randomly fragmented to a size of 350 bp by shearing, then DNA fragments were end polished, A-tailed, and ligated with the NEBNext adapter for Illumina sequencing, and further PCR enriched by P5 and indexed P7 oligos. The PCR products were purified (AMPure XP system) and resultant libraries were analyzed for size distribution by Agilent 2100 Bioanalyzer and quantified using real-time PCR. Sequencing was performed on the Illumina platform (HiSeq X) using a paired-end read length of 150 bp. Data files have been uploaded to the European Nucleotide Archive with accession number PRJEB40922.

### Sequence Alignment and Data Processing

Read pairs with adapter contamination, more than 10% bases uncertainty or >50% low quality bases in either read were first discarded. Burrows-Wheeler Aligner (BWA) was utilized to map the paired-end reads to the human reference genome b37 (ftp.broadinstitute.org/bundle/b37/human_g1k_v37_decoy.fasta.gz) and duplicate reads marked using Picard (http://picard.sourceforge.net) (Li and Durbin, 2009). The BAM files were further processed following the GATK Best Practices Workflow (https://www.broadinstitute.org/gatk/guide/best-practices). Single-sample genotypes were called using GATK HaplotypeCaller (McKenna et al., 2010) followed by hard filtering with the following options: QualByDepth > 2.0, FisherStrand < 60.0, MappingQuality > 40, MappingQualityRankSumTest > − 12.5, ReadPosRankSumTest > − 8.0 and StrandOddsRatio < 3.0. Variants were annotated using ANNOVAR according to the hg19 reference genome (Wang et al., 2010). Downstream analyses were only performed on biallelic SNPs that passed all quality filters above, had less than 10% of genotype missingness, deviation from Hardy-Weinberg equilibrium *p* > 0.001 and minor allele frequency >10%. Genotypic data from myalgia patients, controls and 1,000 Genomes was used in a principal component analysis (PCA) using PLINK 1.9 (Chang et al., 2015) to identify racial stratification in our dataset, and figures were plotted in R.

### Univariate and Single Variant Analysis

Statistical analyses for all clinical parameters (expressed as mean, 95% CI) were performed using R 3.6.1. Fisher’s exact tests were used for categorical variables and t-tests for continuous variables. Unlike the chi-squared test, Fisher’s exact test does not require the expected frequencies of cases and controls to be large, and was the more suitable test given the small sample size in this study. To determine the association of genetic polymorphisms with myalgia, binary logistic regression was performed on the 4,554,532 SNPs with known rs numbers using PLINK 1.9. Additive, dominant and recessive models for genotypes were separately tested. Sex was included as a covariate as it was found to be significantly associated with myalgia, and the first two principal components (PCs) were used to correct for population substructure. SNPs obtained from this single variant analysis were ranked according to the lowest *p*-value out of the three genotypic models tested.

For a 0.1 minor allele frequency cutoff, assuming a reported prevalence rate of 0.2 (prevalence has been reported to be up to 25%) and a case control ratio of 1:5, to detect an odds ratio of 5 with *p* < 5 × 10^−8^ and 80% statistical power, a sample size of 34 cases for the additive model and 35 cases for the dominant model is required. However, the prevalence rate of myalgia in this study may not be 0.2 as there were only 30 patients with myalgia out of the 976 patients on atorvastatin therapy whose clinical data was available. Assuming a prevalence rate of 30/976 = 0.03, 66 cases would be required to detect the above effects.

### Selection of Potentially Functional SNPs

Potential functionalities of SNPs found in this study were evaluated using the pfSNP resource developed by our laboratory (Wang et al., 2011). pfSNPs include SNPs that reside within regions under natural selection forces, as well as those predicted to alter the expression, structure, function, or activity of the associated gene. For coding SNPs, functionality was determined based on whether the SNP resides within protein modification sites such as phosphorylation sites, within important protein domains/functional regions, or are predicted to affect exonic splice enhancer/silencer sites or nonsense-mediated decay. Furthermore, within the coding region, synonymous mutations were assessed for significant codon usage bias as this could potentially influence the speed of the translation process (Kimchi-Sarfaty et al., 2007), while predicted deleteriousness was used for selecting non-synonymous pfSNPs (Bachtiar et al., 2019b). In addition to the pfSNP resource, expression quantitative trait loci (eQTLs) from the GTEx database (gtexportal.org) (Carithers et al., 2015), the eqtlGen consortium (eqtlgen.org) (Võsa et al., 2018) and the Jansen study (eqtl.onderzoek.io) (Jansen et al., 2017) were also used to identify potentially functional SNPs (pfSNPs). Cumulative counts of potentially functional (pf) SNPs were compared with non-pf SNPs for the top 100 SNPs most associated with myalgia based on univariate association *p*-values.

### Selection of Candidate SNPs for Prediction

Three separate groups of SNPs were used as inputs into the machine learning models. These were: 1) SNPs that were most highly associated with myalgia from our results, 2) SNPs residing in 128 genes in the atorvastatin pathway from the drug databases Drugbank, CHEMBL, CTD and PharmGKB as previously obtained by our group (Supplementary Table S1) (Bachtiar et al., 2019b), and 3) SNPs in nine genes reported to be associated with atorvastatin-induced myalgia from the literature (Supplementary Table S2) (Ruano et al., 2007; Ruano et al., 2011; Brunham et al., 2018). SNPs in these three groups were ranked by their *p*-value of association with myalgia from our univariate analysis, and the top 50 overall, pf and non-pf SNPs from these three groups were extracted and separately used for training the models. For genes found to be associated with myalgia from the literature, only 20 non-pf SNPs were found. SNPs with missing values as well as those with greater than 80% correlation with a more significant SNP were removed. Non-pf SNPs that had greater than 80% correlation with pfSNPs were also removed from the non-pf group.

### Predictions Using Machine Learning

Six classifiers were selected for predicting myalgia. These include regression based methods such as logistic regression and elastic nets; tree based methods such as random forests and boosted trees and other popular machine learning approaches such as neural networks and support vector machines. As there is currently no consensus as to which approach is best for genomic data, these six models were selected as a broad representation of popular machine learning models used for prediction. SNPs that performed well on most or all models represent SNPs that are able to predict myalgia to a high degree of confidence. As the different models use different approaches for learning and prediction, consistent results from the majority of models would increase our confidence about the validity of the results. All predictions were made using the R caret package in conjunction with the glm, glmnet, rf, gbm, nnet and svmRadial packages for training the individual models (Kuhn, 2008). Default caret training settings were used and sex was included as a predictor in all models. Predictive performance using the top 5–50 (in intervals of 5) overall, pf and non-pf SNPs from all three groups were separately obtained using the average 5-round 5-fold cross validation area under ROC curve (AUC) as the performance score. The unpaired t-test with Bonferroni correction (*n* = 3) was used to determine if there was a significant difference in mean AUC values of models using pfSNPs, non-pf SNPs and all SNPs. All six models were also trained without SNP data using 1) only sex as a predictor and 2) all clinical characteristics as predictors for determining the baseline model.

## Results

### Demographic and Clinical Characteristics

There were 88 Chinese, 57 Indians and 38 Malays in the dataset and patients ranged in age from 25 to 81 years (mean = 57.4, CI = 55.9–59.0). The ethnic distribution in the study cohort is generally reflective of the Singapore population, although there was a lower percentage of Chinese and a higher percentage of Indians in the study cohort. This can be attributed to the higher prevalence of coronary heart disease in Singapore Indians requiring statin pharmacotherapy resulting in a higher proportion of Indians among statin users (Hughes et al., 1990; Ounpuu and Yusuf, 2003). All patients were treated with atorvastatin and the demographic and clinical characteristics of patients according to myalgia status is shown in Table 1. Of these characteristics, only sex was found to be significant (*p* < 0.05), with females more likely to have statin induced myopathy than males (Table 1). None of the comorbidities and drug treatments were found to be significantly associated with myalgia.

**TABLE 1:** Clinical/demographic characteristics of myalgia (cases) and non-myalgia (controls) subjects.

Characteristic	Descriptor	Group		*p*
		Myalgia *n* = 30	Non myalgia *n* = 153	
Age (yrs)^a^		56.7 (52.6–60.8)	57.6 (55.9–59.2)	0.71
BMI (kg/m^2^)		27.3 (25.5–29.1)	26.3 (25.6–27.0)	0.29
Statin dose (mg)		36.0 (28.7–43.3)	38.4 (35.4–41.5)	0.54
Days on statin		702 (344–1,060)	805 (657–953)	0.59
Reported ethnicity	Chinese	14 (46.7%)	74 (48.4%)	0.44
	Indian	12 (40.0%)	45 (29.4%)	
	Malay	4 (13.3%)	34 (22.2%)	
Sex	Male	22 (73.3%)	136 (88.9%)	0.038
	Female	8 (26.7%)	17 (11.1%)	
Alcohol consumption	No	27 (90.0%)	141 (92.2%)	0.72
	Yes	3 (10.0%)	12 (7.8%)	
Smoking	No	25 (83.3%)	119 (77.8%)	0.63
	Yes	5 (16.7%)	34 (22.2%)	
Myocardial infarction	No	6 (20%)	12 (7.8%)	0.085
	Yes	24 (80%)	141 (92.2%)	
Renal problems	No	23 (76.7%)	132 (86.3%)	0.18
	Yes	7 (23.3%)	21 (13.7%)	
Liver problems	No	28 (93.3%)	146 (95.4%)	0.64
	Yes	2 (6.7%)	7 (4.6%)	
Hypertension	No	13 (43.3%)	57 (37.3%)	0.54
	Yes	17 (56.7%)	96 (62.7%)	
Diabetes mellitus	No	20 (66.7%)	82 (53.6%)	0.23
	Yes	10 (33.3%)	71 (46.4%)	
Hypercholesterolemia	No	11 (36.7%)	44 (28.8%)	0.39
	Yes	19 (63.3%)	109 (71.2%)	
Blood thinner	No	5 (16.7%)	15 (9.8%)	0.33
	Yes	25 (83.3%)	138 (90.2%)	
Glucose lowering	No	21 (70%)	78 (51%)	0.071
	Yes	9 (30%)	75 (49%)	
Cholesterol lowering	No	28 (93.3%)	133 (86.9%)	0.54
	Yes	2 (6.7%)	20 (13.1%)	
Heart protective	No	19 (63.3%)	74 (48.4%)	0.16
	Yes	11 (36.7%)	79 (51.6%)	
Blood pressure lowering	No	23 (76.7%)	91 (59.5%)	0.099
	Yes	7 (23.3%)	62 (40.5%)	

^a^ One sample in the non-myalgia group was missing age data.

### Population Stratification

PCA analyses showed that Chinese patients from our dataset clustered more closely with 1,000 Genomes East Asian populations, and Indian patients from our dataset clustered more closely with 1,000 Genomes South Asian populations (Supplementary Figure S1A). Chinese, Malay and Indian patients from our dataset were also fairly well separated when projected on to the first two principal components (Supplementary Figure S1B), although there was some overlap between Chinese and Malay patients due to genetic admixture between the two ethnicities (Deng et al., 2015).

### Single Variant Analyses

**FIGURE 1:**
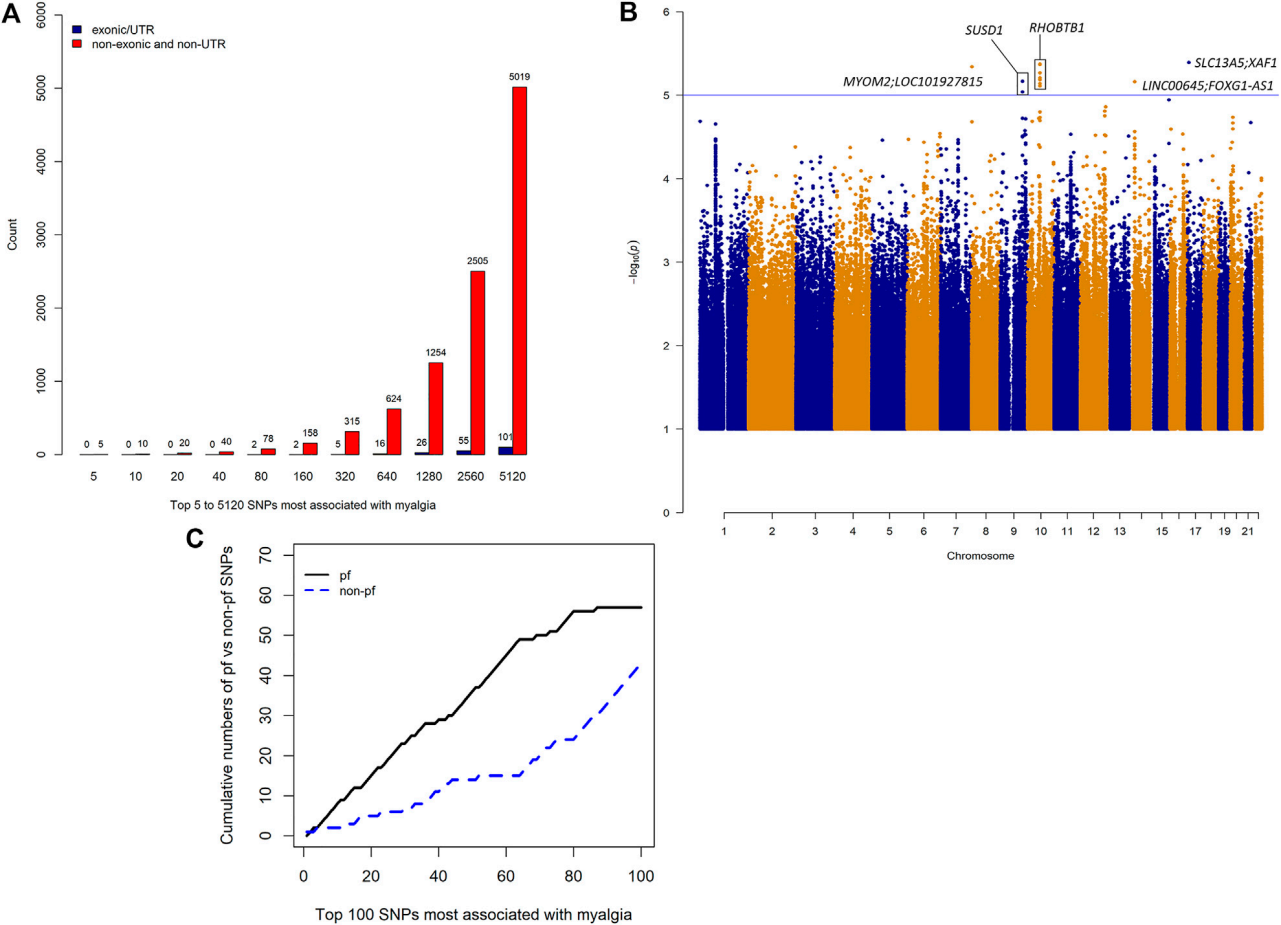
Whole genome sequencing results. **(A)** Number of exonic/UTR variants vs non-exonic/non-UTR variants in the top 5 to 5,120 SNPs most associated with myalgia. **(B)** Manhattan plot of association of SNPs with atorvastatin-induced myalgia. The line indicates a *p*-value threshold of 1 × 10^−5^, which can be considered to be a suggestive threshold of genome wide significance for small sample sizes. **(C)** Cumulative numbers of potentially functional (pf) and non-pf SNPs in the top 100 SNPs most associated with myalgia.

4,554,532 SNPs with known rs numbers passed quality control in our dataset, with the majority of variants residing in intergenic and intronic regions (Supplementary Figure S2). To identify single SNP variants that might be associated with statin induced myalgia, logistic regression adjusting for the first two principal components and sex was performed. Most of the SNPs that were highly associated with myalgia were located outside exons and untranslated (UTR) regions (Figure 1A), highlighting an important limitation of exome based platforms. A *p*-value of 5 × 10^−8^ is commonly used to determine significance in genome wide studies, based on an assumption of 1,000,000 independent tests and patterns of linkage disequilibrium in individuals of European descent (Fadista et al., 2016). Although none of the variants in our analyses met this *p*-value threshold, 15 suggestive SNPs (*p* < 1 × 10^−5^) were found, with genes *RHOBTB1* on chromosome 10 and *SUSD1* on chromosome 9 containing the most number of suggestive SNPs, all of which were potentially functional (Figure 1B; Table 2). The top SNP for *RHOBTB1*, rs10821852, is an intronic SNP with an odds ratio (OR) of 5.66 (95% CI: 2.70–11.8, *p*: 4.23 × 10^−6^, assuming an additive genotypic model) while the top SNP for *SUSD1*, rs10981237 is an intronic SNP with an OR of 21.67 (95% CI: 5.68–82.8, *p*: 6.81 × 10^−6^, assuming a recessive genotypic model) (Table 2).

**TABLE 2:** Top single variant associations with atorvastatin-induced myalgia (*p* < 1 × 10^−5^).

rsID	Chr	BP	OR (CI)	*p*	Model	Location	Gene	Intergenic distances	pfSNP
rs8082182	17	6,622,978	17.87 (5.244–60.91)	4.05*E* − 06	REC	Intergenic	*SLC13A5*; *XAF1*	dist = 6,238; dist = 36,178	NA
rs10821852	10	62,660,939	5.658 (2.704–11.84)	4.23*E* − 06	ADD	Intronic	*RHOBTB1*		eQTL
rs12263661	10	62,661,320	6.321 (2.88–13.87)	4.27*E* − 06	ADD	Intronic	*RHOBTB1*		eQTL
rs7011427	8	2,152,675	8.345 (3.369–20.67)	4.54*E* − 06	REC	Intergenic	*MYOM2*; *LOC101927815*	dist = 59,295; dist = 234,544	NA
rs750593	10	62,662,314	6.097 (2.798–13.28)	5.39*E* − 06	ADD	Intronic	*RHOBTB1*		eQTL
rs10821851	10	62,660,911	5.145 (2.529–10.47)	6.16*E* − 06	ADD	Intronic	*RHOBTB1*		eQTL
rs4437981	10	62,662,503	6.013 (2.758–13.11)	6.45*E* − 06	ADD	Intronic	*RHOBTB1*		eQTL
rs4575214	10	62,662,468	6.013 (2.758–13.11)	6.45*E* − 06	ADD	Intronic	*RHOBTB1*		eQTL
rs2893868	10	62,661,125	5.998 (2.753–13.07)	6.50*E* − 06	ADD	Intronic	*RHOBTB1*		eQTL
rs10981237	9	1,14,817,524	21.67 (5.675–82.76)	6.81*E* − 06	REC	Intronic	*SUSD1*		eQTL
rs16916623	9	114,821,568	21.67 (5.675–82.76)	6.81*E* − 06	REC	Intronic	*SUSD1*		eQTL
rs8011850	14	29,117,256	9.648 (3.593–25.91)	6.87*E* − 06	ADD	Intergenic	*LINC00645*; *FOXG1-AS1*	dist = 1,008,414; dist = 77,192	NA
rs2893869	10	6,2,661,961	5.787 (2.688–12.46)	7.25*E* − 06	ADD	Intronic	*RHOBTB1*		eQTL
rs10821853	10	62,661,057	5.286 (2.548–10.97)	7.74*E* − 06	ADD	Intronic	*RHOBTB1*		eQTL
rs55744607	9	114,815,563	17.78 (4.986–63.37)	9.11*E* − 06	REC	Intronic	*SUSD1*		eQTL

### Distribution of Potentially Functional SNPs

Of the 4,554,532 SNPs with known rs numbers, approximately 60% (2,774,804) were potentially functional. The cumulative number of pfSNPs was consistently higher than that of non-pf SNPs in the top 100 SNPs most associated with myalgia (Figure 1C).

### Good Predictive Performance Using 15 SNPs

**FIGURE 2:**
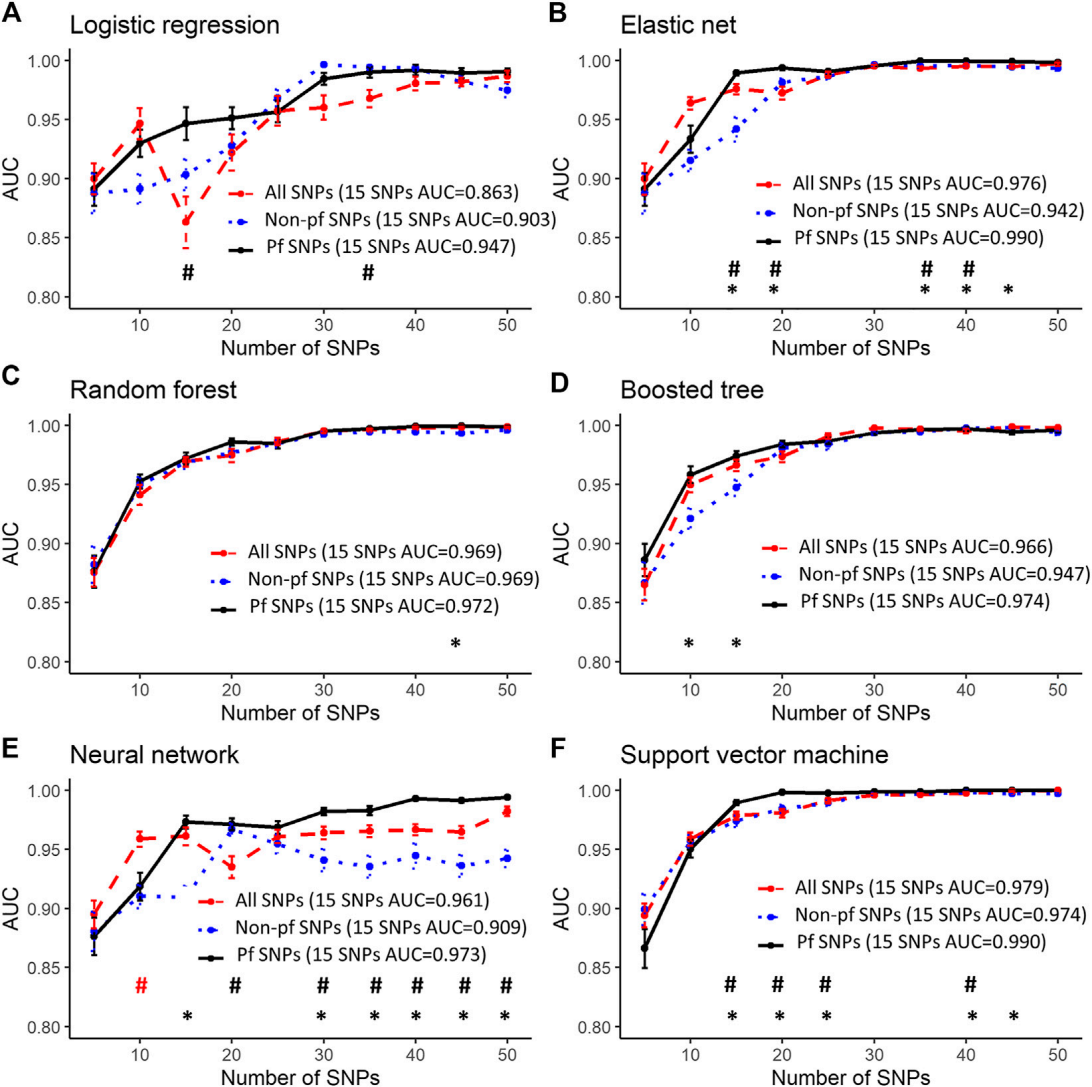
Predictive performance using the top 5 to 50 SNPs most associated with myalgia from our dataset. Error bars denote the standard error of the mean. #’s indicate statistical significance when comparing between all SNPs and pfSNPs while *’s indicate statistical significance when comparing between non-pf SNPs and pfSNPs. Statistical significance when comparing between all SNPs and non-pfSNPs is not shown. The colors represent the input set with the higher AUC (red—all SNPs, blue—non-pf SNPs and black—pfSNPs). Bonferroni corrected unpaired *t*-test *p*-values (*p* < 0.05) were used for determining statistical significance.

**FIGURE 3:**
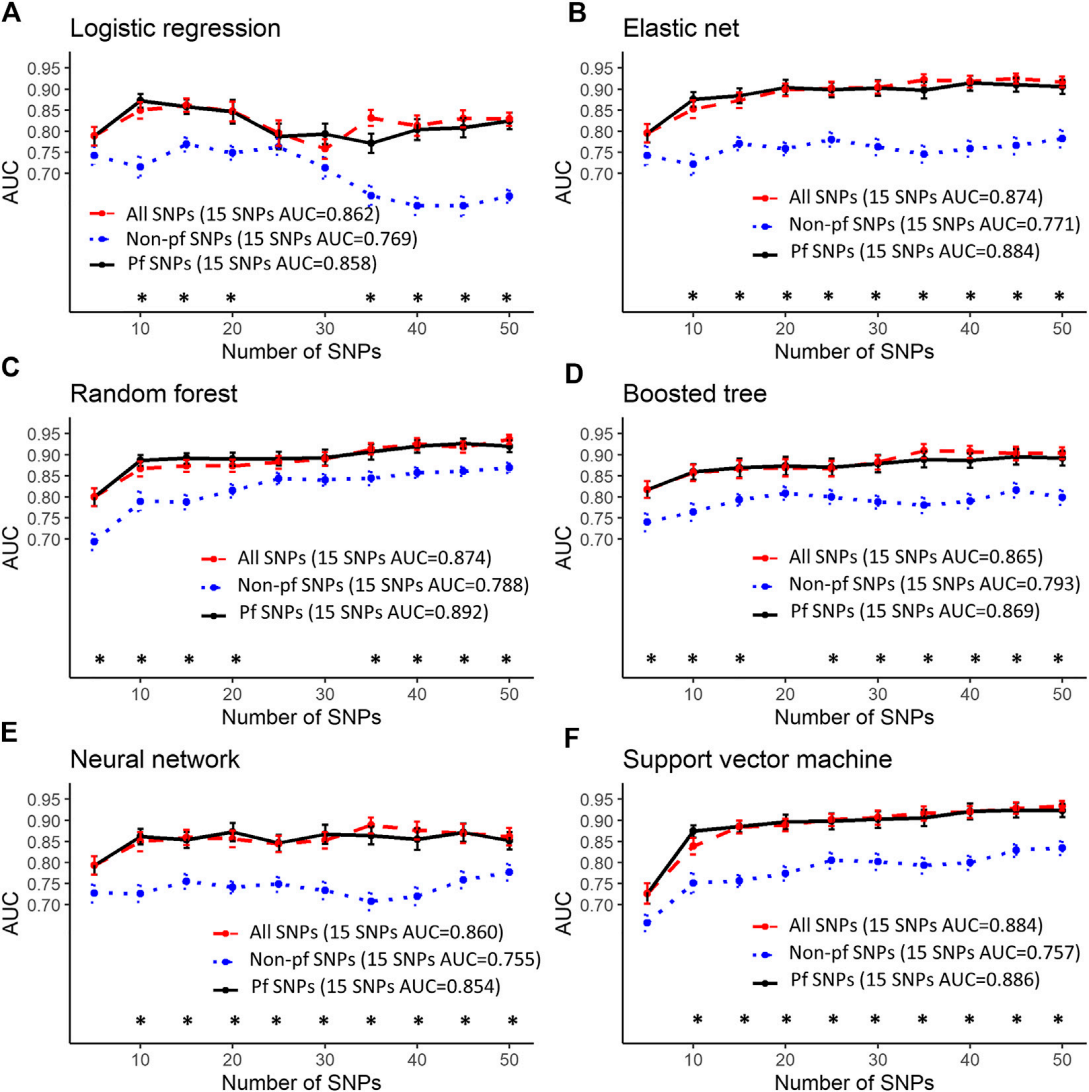
Predictive performance using the top 5–50 SNPs in atorvastatin pathway genes. Error bars denote the standard error of the mean. #’s indicate statistical significance when comparing between all SNPs and pfSNPs while *’s indicate statistical significance when comparing between non-pf SNPs and pfSNPs. Statistical significance when comparing between all SNPs and non-pfSNPs is not shown. The colors represent the input set with the higher AUC (red—all SNPs, blue—non-pf SNPs and black—pfSNPs). Bonferroni corrected unpaired *t*-test *p*-values (*p* < 0.05) were used for determining statistical significance.

**FIGURE 4:**
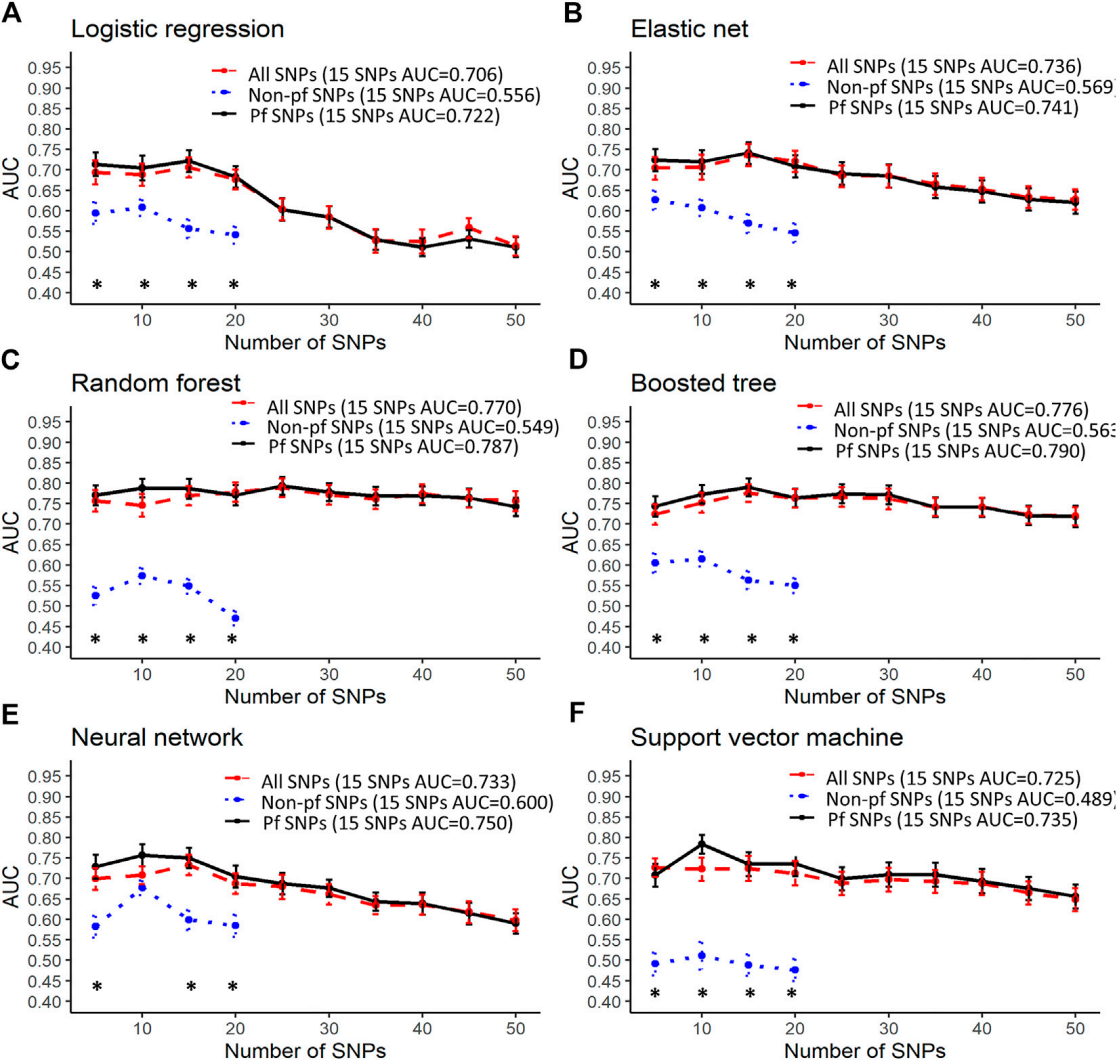
Predictive performance using the top 5–50 SNPs in genes found to be associated with myalgia from previous studies. Error bars denote the standard error of the mean. #’s indicate statistical significance when comparing between all SNPs and pfSNPs while *’s indicate statistical significance when comparing between non-pf SNPs and pfSNPs. Statistical significance when comparing between all SNPs and non-pfSNPs is not shown. The colors represent the input set with the higher AUC (red—all SNPs, blue—non-pf SNPs and black—pfSNPs). Bonferroni corrected unpaired *t*-test *p*-values (*p* < 0.05) were used for determining statistical significance. Only 20 non-pf SNPs were found in genes associated with myalgia from the literature.

Predictive performance was greatest when using SNPs that were highly associated with myalgia from this study (highest AUC: 1, Figure 2) followed by SNPs in atorvastatin pathway genes (highest AUC: 0.936, Figure 3) and SNPs in myalgia associated genes from previous studies (highest AUC: 0.794, Figure 4). For all models and inputs, close to maximal AUCs were generally achieved when 15 SNPs were used, after which there was either minimal increase in predictive performance, or a decrease in AUC values (Figures 2–4). However, for SNPs in myalgia associated genes from previous studies, mean AUC values did not increase with increasing number of SNPs, suggesting that most of these SNPs were not predictive (Figure 4). Out of the top five pfSNPs in this group, four were within the *ABCG2* gene while one was at the *HTR3B* locus. In terms of the best performing machine learning model when 15 SNPs were used as inputs, the best model was support vector machine (AUC: 0.990) for SNPs found from this study (Figure 2), random forest (AUC: 0.89) for atorvastatin pathway SNPs (Figure 3) and boosted tree (AUC:0.790) for SNPs in genes from previous studies (Figure 4).

### Robust Performance of Potentially Functional SNPs in Predicting Myalgia

The best performing models described above for a 15 SNP input were obtained when using only pfSNPs, and not when using all SNPs or non-pf SNPs. Furthermore, when comparing pfSNPs to combined SNPs, in four of the machine learning models (logistic regression, elastic net, neural network, support vector machine), pfSNPs outperformed combined SNPs when 15 or more SNPs were used (Figures 2A, B, E, F, # indicates Bonferroni corrected *p* < 0.05). The predictive performance of pfSNPs was only significantly lower than the combined SNPs when a small number of 10 SNPs was used in the neural network model (Figure 2E, # indicates Bonferroni corrected *p* < 0.05). However, the AUC achieved using this 10 combined SNPs was 0.959 which was lower than when 15 pfSNPs were used in the same neural network model (AUC: 0.973). In the remaining models (Figures 2C,D) as well as for atorvastatin SNPs (Figure 3) and literature review SNPs (Figure 4), there was no significant difference between using pfSNPs and using combined SNPs. When comparing pfSNPs to non-pf SNPs, pfSNPs outperformed non-pf SNPs in almost all models and input sets (Figures 2–4, * indicates Bonferroni corrected *p* < 0.05). Additionally, the baseline performance of models only incorporating sex as a predictor (best AUC: 0.58) or using all clinical variables (best AUC: 0.57) (Supplementary Table S3) was significantly poorer than models incorporating both pfSNPs and sex (Figures 2–4).

## Discussion

In this study, we hypothesize that rather than individual SNPs, a combination of several potentially functional SNPs (pfSNPs) can better predict myalgia in patients on atorvastatin. Among the demographic and clinical characteristics examined, only sex was significantly (*p* < 0.05) associated with myalgia, with females having a higher risk. This is concordant with reports from previous studies (Link et al., 2008; Bakar et al., 2018; Tournadre, 2020). Through whole genome association analyses with sex as a covariate, we first demonstrated that among the top 100 SNPs that were most associated with myalgia, the cumulative number of pfSNPs was consistently higher than that of non-pf SNPs (Figure 1C) highlighting the importance of pfSNPs. To identify the combination of pfSNPs/non-pfSNPs that can predict atorvastatin-induced myalgia, six different, but commonly used machine learning models were employed to identify the minimum number of pfSNPs/non-pfSNPs necessary to achieve optimal sensitivity and specificity, determined through the area under ROC curve (AUC), in most, if not all the six models. pfSNPs consistently outperforms non-pfSNPs in predicting myalgia. To our knowledge, this is the first study examining pfSNPs and utilizing machine learning models in the prediction of myalgia.

From the whole genome sequencing results, potentially functional SNPs in *RHOBTB1* and *SUSD1* were found to be highly associated with atorvastatin-induced myalgia. *RHOBTB1* is a member of the Rho GTPase family of signaling proteins with high levels of expression in the stomach, skeletal muscle, placenta, kidney and testis (Ramos et al., 2002). *RHOBTB1* is a tumor suppressor gene involved in head and neck cancer and is also involved in protecting against hypertension by improving vasodilator function (Xiao et al., 2017; Mukohda et al., 2019). Knockdown of *RHOBTB1* was also found to promote cardiomyocyte proliferation (Xiao et al., 2017). Given its high expression in skeletal muscle, as well as its role in cardiomyocyte proliferation and preventing vascular smooth muscle dysfunction, it is possible that this gene is also involved in preventing myalgia. Not much is known about *SUSD1*, which encodes for the sushi domain-containing protein 1 precursor. The sushi domain has been found in a number of proteins and is a motif for protein-protein interactions (Wei et al., 2001). SNPs in *SUSD1* has been previously associated with venous thromboembolism (Tang et al., 2013) and neurocognitive disabilities (Nilsson et al., 2017).

Most of the machine learning models gave similar AUC values making it difficult to draw definitive conclusions as to which model performs best. However, models including only clinical factors (Supplementary Table S3) were found to have a poorer performance than models incorporating sex and genetic factors (Figures 2–4), demonstrating the higher predictive potential of SNPs compared to clinical factors. When 15 or more SNPs were used, elastic net, neural network and support vector machine models with potentially functional SNPs as inputs had significantly better mean AUCs compared to the same models incorporating non-pf SNPs and total SNPs as seen in Figure 2. Furthermore, the overall best models at the 15 SNP level for each of the three datasets (associated SNPs from this study, SNPs in artovastatin pathway genes and SNPs in genes found from the literature) all utilized pfSNPs as inputs (Figures 2–4). Taken together, these results suggest that SNP functionality is an important factor to consider for improving predictive performance. The importance of SNP functionality was also underscored by the fact that the raw count of pfSNPs was higher than non-pf SNPs in the top 100 variants most associated with myalgia.

Interestingly, we found that SNPs in genes previously reported to be associated with myalgia had the poorest predictive performance of the three groups. Furthermore, predictive AUCs in this group did not increase as the number of SNPs used was increased. Genes in this group include the serotonin receptor genes *HTR3B* and *HTR7* (Ruano et al., 2007), efflux transporter *ABCG2*, uptake transporter *SLCO1B1*, cytochrome P450 genes *CYP3A4* and *CYP2D6*, and other candidate genes such as *COQ2*, *ATP2B1*, *DMPK* (Ruano et al., 2011). Our results suggest that only a few SNPs in this group had predictive value, with *ABCG2* and *HTR3B* being the strongest candidate genes. It is also interesting to note that the rs4149056 variant in the *SLCO1B1* gene had a relatively high uncorrected *p*-value of 0.1 in our study. Furthermore, the minor allele frequencies of this variant were higher in controls than in cases for Singaporeans of Chinese, Malay and Indian ethnicities (Supplementary Table S4). These findings suggest that the rs4149056 variant may not have the same effect for milder myalgia, in non-European populations, or due to the type of statin used. These reasons were also alluded to in the review by Turner and Pirmohamed (2019) when discussing the role of *SLCO1B1* in statin-related myotoxicity.

There are however some limitations to this study. The relatively small number of samples, with only 30 patients reporting definitive myalgia, limits the discovery *p*-value to only a suggestive threshold, and could be a possible reason why *SLCO1B1* was not detected to be significant. Nevertheless, smaller sample sizes are not unusual in pharmacogenomic association studies due to the large effect sizes of pharmacogenomic variants, unlike complex disease association analyses (Maranville and Cox, 2016). Furthermore, in this study, being unable to achieve genome wide significance for single SNPs is not pertinent as the univariate *p*-values were merely used for the ranking of SNPs to facilitate the identification of a combination of multiple potentially functional SNPs that best predict atorvastatin-induced myalgia using six different machine learning algorithms. The combination of pfSNPs that were found by most, if not all, of the six different machine learning models to show high sensitivity and specificity in predicting myalgia highlights the robustness of our strategy. A second caveat is that predictive performance of the machine learning models, while achieving good cross validation AUCs, should ideally be validated against an independent test set. Nonetheless, cross validation is a useful indicator of the generalizability of the model and by utilizing the lowest number of SNPs with good AUCs, which we found to be in the 15 SNP range, we hope to minimize overfitting. We aim to validate these SNPs in an independent test set in a future study. The results of this study, while limited by the small sample size, represent a proof of concept of the potential of both machine learning methods and potentially functional polymorphisms in the prediction of drug response.

In conclusion, machine learning models with potentially functional SNPs were found to have good and robust properties for predicting atorvastatin-induced myalgia. However, SNPs in candidate genes previously reported to be associated with myalgia did not show good predictive properties, at least in this Singapore population. Combinations of pfSNPs that were consistently identified by different machine learning models to have high predictive performance have good potential to be clinically useful for predicting atorvastatin-induced myalgia once validated against an independent cohort of patients.

## Data Availability

The datasets presented in this study can be found in online repositories. The names of the repository/repositories and accession number(s) can be found below: https://www.ebi.ac.uk/ena, PRJEB40922.
