# Supplementary material for: Robust Performance of Potentially Functional SNPs in Machine Learning Models for the Prediction of Atorvastatin-Induced Myalgia
Source: Front Pharmacol. 2021 Apr 22;12:605764. doi: 10.3389/fphar.2021.605764 (PMC8100589; doi:10.3389/fphar.2021.605764)
Supplement: Supplementary file 1 [file datasheet1.docx]

**Supplementary Information:**

**Robust performance of potentially functional SNPs in machine learning models for the prediction of atorvastatin-induced myalgia**

This file includes:

**Supplementary Figure 1.** Principal components analysis.

**Supplementary Figure 2.** Distribution of SNPs that passed quality control from whole genome sequencing.

**Supplementary Table 1.** List of genes from the drug databases Drugbank, CHEMBL, CTD and PharmGKB.

**Supplementary Table 2.** List of genes reported to be associated with atorvastatin-induced myalgia from previous studies in the literature

**Supplementary Table 3.** Predictive performance of baseline machine learning models using either all clinical/demographic characteristics or gender

**Supplementary Table 4.** Minor allele frequencies of the SLCO1B1 variant rs4149056 in Singaporean Chinese, Malay and Indian compared to European ethnicities

**
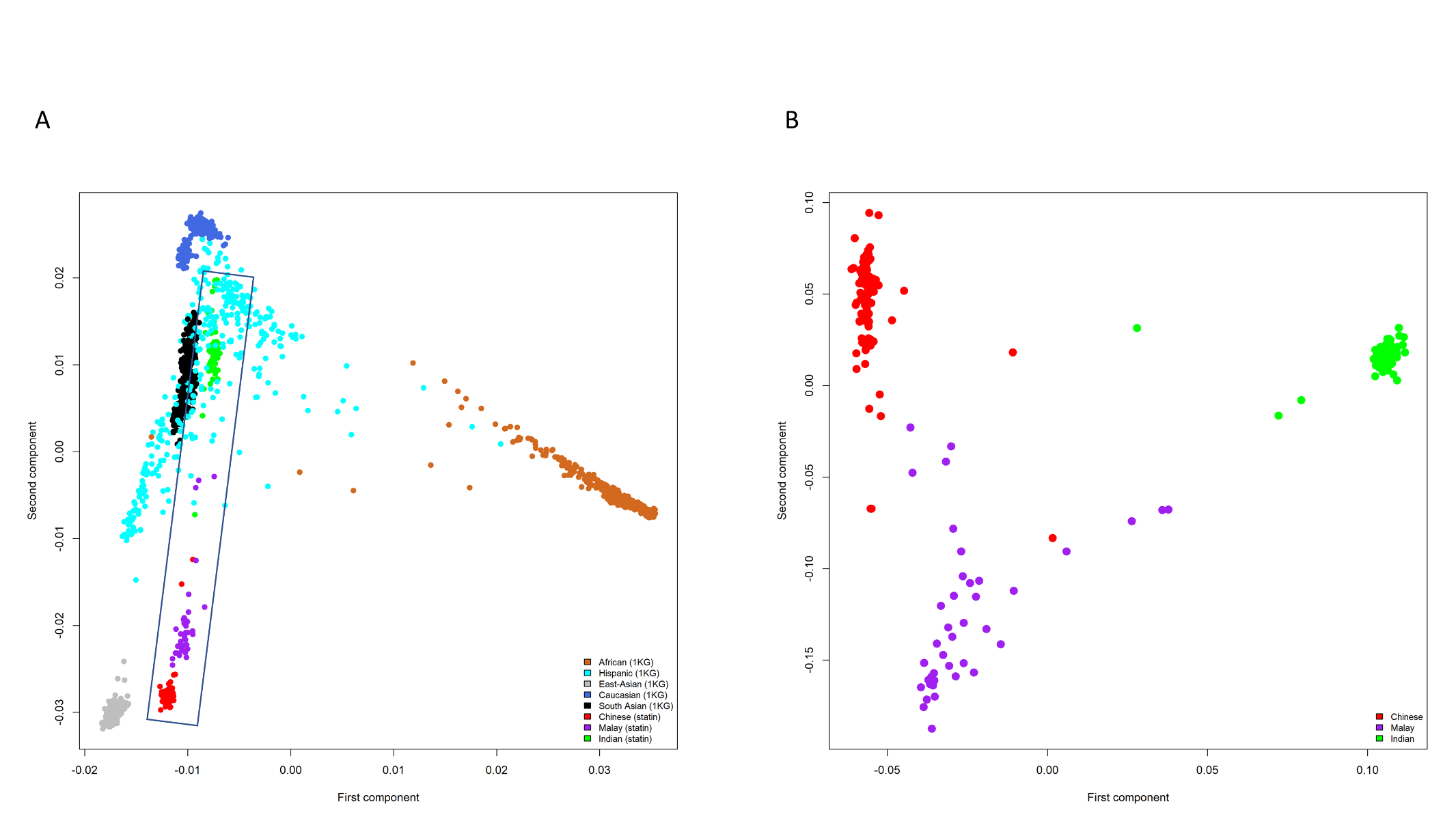
**

**Supplementary Figure 1**. **Principal components analysis**. (A) Plot of the first two principal components of variation based on combined 1000 Genomes and atorvastatin dataset (B) Plot of the first two principal components of variation based only on the atorvastatin dataset

**
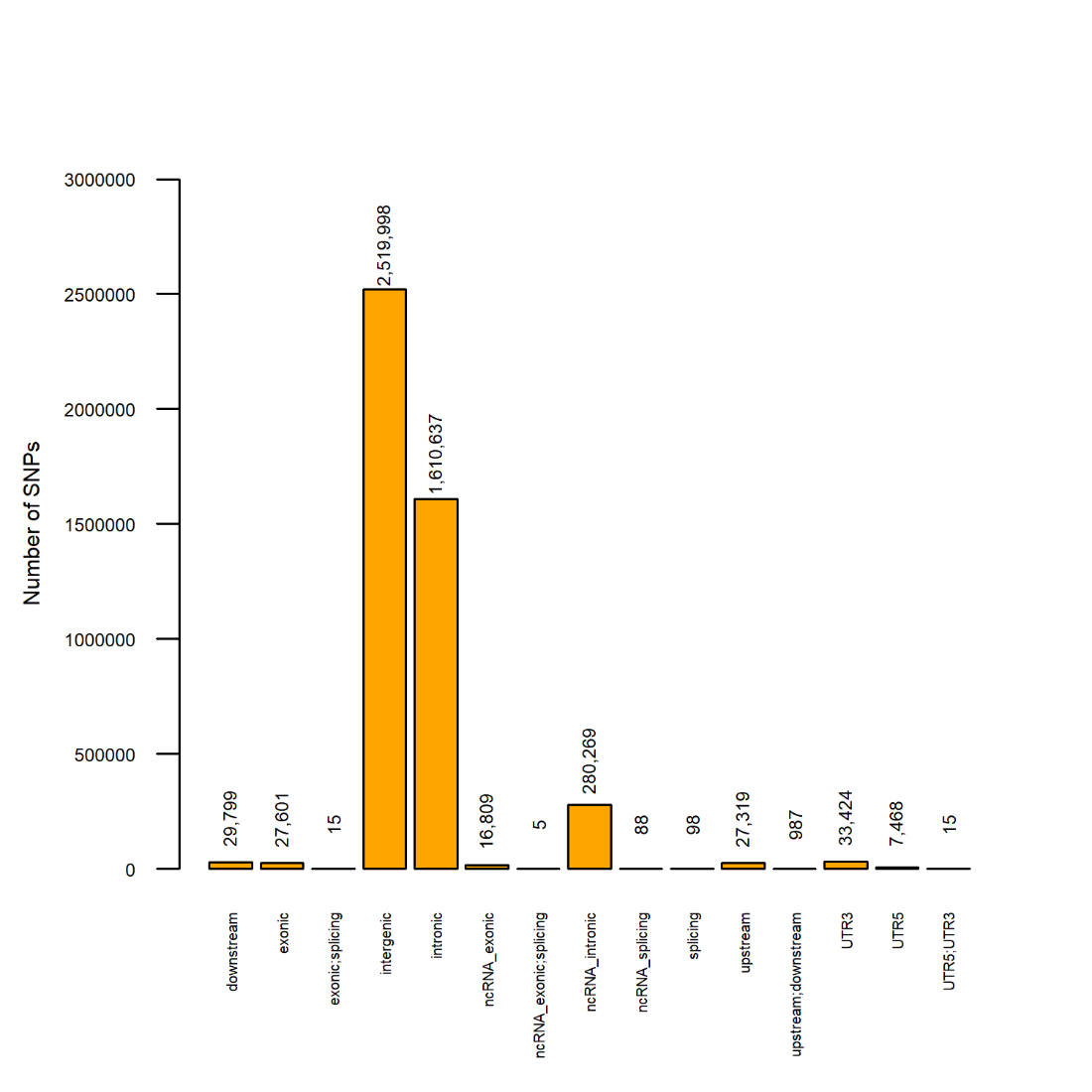
**

**Supplementary Figure 2: Distribution of SNPs that passed quality control from whole genome sequencing**

**Supplementary Table 1**: List of genes from the drug databases Drugbank, CHEMBL, CTD and PharmGKB

| **Gene** | **Database** |
| --- | --- |
| *ABCA1* | PHARMGKB, CTD |
| *ABCB1* | PHARMGKB, CTD |
| *ABCB11* | PHARMGKB, CTD |
| *ABCC2* | PHARMGKB, CTD |
| *ABCG2* | PHARMGKB |
| *ABCG5* | PHARMGKB |
| *ABCG8* | PHARMGKB |
| *ADIPOQ* | CTD |
| *AHR* | DRUGBANK |
| *AKT1* | CTD |
| *ANGPT2* | CTD |
| *APOA1* | PHARMGKB |
| *APOA4* | PHARMGKB |
| *APOA5* | PHARMGKB |
| *APOB* | PHARMGKB, CTD |
| *APOC1* | PHARMGKB |
| *APOC2* | PHARMGKB |
| *APOC3* | PHARMGKB |
| *APOE* | PHARMGKB, CTD |
| *BAK1* | CTD |
| *BAX* | CTD |
| *BCL2* | CTD |
| *BGLAP* | CTD |
| *BMP2* | CTD |
| *CASP3* | CTD |
| *CASP8* | CTD |
| *CASP9* | CTD |
| *CAT* | CTD |
| *CCL2* | CTD |
| *CD14* | CTD |
| *CD36* | CTD |
| *CD40* | CTD |
| *CD40LG* | CTD |
| *CD83* | CTD |
| *CD86* | CTD |
| *CDKN1A* | CTD |
| *CDKN1B* | CTD |
| *CETP* | PHARMGKB |
| *COL1A1* | CTD |
| *CRP* | CTD |
| *CYP27A1* | CTD |

| **Gene** | **Database** | |
| --- | --- | --- |
| *CYP2B6* | CTD | |
| *CYP2C19* | PHARMGKB | |
| *CYP2C8* | PHARMGKB | |
| *CYP2C9* | PHARMGKB | |
| *CYP2D6* | PHARMGKB | |
| *CYP3A4* | PHARMGKB, CTD | |
| *CYP3A5* | PHARMGKB | |
| *CYP7A1* | PHARMGKB | |
| *DGAT1* | PHARMGKB | |
| *DPP4* | DRUGBANK | |
| *EDN1* | CTD | |
| *EIF2AK3* | CTD | |
| *EIF2S1* | CTD | |
| *F2* | CTD | |
| *F5* | CTD | |
| *FASLG* | CTD | |
| *FASN* | CTD | |
| *FDFT1* | PHARMGKB | |
| *FIGF* | CTD | |
| *HMGCR* | PHARMGKB, CTD, DRUGBANK, CHEMBL_13 | |
| *HMOX1* | CTD | |
| *ICAM1* | CTD | |
| *IL12A* | CTD | |
| *IL2RA* | CTD | |
| *IL6* | CTD | |
| *IL8* | CTD | |
| *INS* | CTD | |
| *IRAK1* | CTD | |
| *ITGAL* | CTD | |
| *ITGAM* | CTD | |
| *ITGAX* | CTD | |
| *ITGB2* | CTD | |
| *JUN* | CTD | |
| *KIF6* | CTD | |
| *LCAT* | PHARMGKB | |
| *LDLR* | PHARMGKB, CTD | |
| *LIPC* | PHARMGKB | |
| *LPL* | PHARMGKB | |
| *LRP1* | PHARMGKB | |
| *MAPK1* | CTD | |
| *MAPK14* | CTD | |
| *MAPK3* | CTD | |
| *MCL1* | CTD | |
| *MMP9* | CTD | |
| *MSR1* | CTD | |
| **Gene** | | **Database** |
| *MTTP* | | CTD |
| *NOS3* | | CTD |
| *NOX4* | | CTD |
| *NR1I3* | | CTD |
| *PARP1* | | CTD |
| *PDPK1* | | CTD |
| *PLAU* | | CTD |
| *PLTP* | | PHARMGKB |
| *PON1* | | CTD |
| *PON2* | | CTD |
| *PPARA* | | CTD |
| *RAC1* | | CTD |
| *RB1* | | CTD |
| *RHOA* | | CTD |
| *RHOB* | | CTD |
| *RHOC* | | CTD |
| *SCARB1* | | PHARMGKB, CTD |
| *SELE* | | CTD |
| *SLC15A1* | | PHARMGKB |
| *SLC22A1* | | CTD |
| *SLC22A3* | | CTD |
| *SLC22A5* | | CTD |
| *SLC22A6* | | PHARMGKB |
| *SLC22A8* | | PHARMGKB |
| *SLCO1B1* | | PHARMGKB, CTD |
| *SLCO1B3* | | PHARMGKB |
| *SLCO2B1* | | PHARMGKB |
| *SOAT1* | | PHARMGKB |
| *SQLE* | | PHARMGKB |
| *SREBF2* | | CTD |
| *TFRC* | | CTD |
| *THBS1* | | CTD |
| *TLR2* | | CTD |
| *TLR4* | | CTD |
| *TNF* | | CTD |
| *TNFRSF1A* | | CTD |
| *TRAF6* | | CTD |
| *UGT1A1* | | PHARMGKB |
| *UGT1A3* | | PHARMGKB |
| *UGT2B7* | | PHARMGKB |
| *VCAM1* | | CTD |
| *VWF* | | CTD |

**Supplementary Table 2**: List of genes reported to be associated with atorvastatin-induced myalgia from previous studies in the literature

| **Gene symbol** | **Reference** | **rsid** | **Reported *p*** | ***p*** |
| --- | --- | --- | --- | --- |
| *ABCG2* | Mirosevic et al. ABCG2 gene polymorphisms as risk factors for atorvastatin adverse reactions: a case-control study. Pharmacogenomics 2015;16:803–815. | rs2231142 | 0.016 | 0.3 |
| *ATP2B1* | Ruano et al. Mechanisms of statin-induced myalgia assessed by physiogenomic associations. Atherosclerosis 2011;218, 451-456 | rs17381194 | < 0.00079 | 0.85 |
| *COQ2* | Ruano et al. Mechanisms of statin-induced myalgia assessed by physiogenomic associations. Atherosclerosis 2011;218, 451-456 | rs4693570 | < 0.000041 | 0.26 |
|  | Oh et al. Genetic determinants of statin intolerance. Lipids Health Dis 2007; 6:7. | unknown |  |  |
| *CYP2D6* | Frudakis et al. CYP2D6*4 polymorphism is associated with statin-induced muscle effects. Pharmacogenet Genomics 2007;17:695–707 | rs3892097 | 0.004 | N.A. |
| *CYP3A4* | Becker et al. Influence of genetic variation in CYP3A4 and ABCB1 on dose decrease or switching during simvastatin and atorvastatin therapy. Pharmacoepidemiol Drug Saf 2010;19:75–81 | rs2740574 | 0.023 | 0.9 |
| *DMPK* | Ruano et al. Mechanisms of statin-induced myalgia assessed by physiogenomic associations.  Atherosclerosis 2011;218:451-456 | rs672348 | < 0.0016 | 0.49 |
| *HTR3B* | Ruano et al. Physiogenomic Association of Statin-related Myalgia to Serotonin Receptors.  Muscle Nerve 2007;36:329-335 | rs2276307 | 0.007 | 0.0023 |
| *HTR7* | Ruano et al. Physiogenomic Association of Statin-related Myalgia to Serotonin Receptors. Muscle Nerve 2007:36, 329-335 | rs1935349 | 0.026 | 0.4 |
| *SLCO1B1* | Voora et al. The SLCO1B1*5 genetic variant is associated with statin-induced side effects. J Am Coll Cardiol 2009;54:1609–1616. | rs4149056 | ≤ 0.03 | 0.1 |
|  | Donnelly et al. Common nonsynonymous substitutions in SLCO1B1 predispose to statin intolerance in routinely treated individuals with type 2 diabetes: a go-DARTS study. Clin Pharmacol Ther 2011;89:210–216 | rs4149056  rs2306283 | 0.043  0.026 | 0.1  0.7 |
|  | Bakar et al. Genetic and clinical factors are associated with statin-related myotoxicity of moderate severity: a case control study. Clin Pharmacol Ther 2018;104:178-187 | rs4149056 | 0.010 | 0.1 |
|  |  |  |  |  |

**Supplementary Table 3.** Predictive performance of baseline machine learning models using either all clinical/demographic characteristics or gender

|  | **AUC ± SEM** | |
| --- | --- | --- |
|  | **All clinical/demographic characteristics*** | **Gender only** |
|  |  |  |
| Logistic regression | 0.51 ± 0.02 | 0.58 ± 0.02 |
| Elastic net | 0.53 ± 0.02 | NA |
| Random forest | 0.54 ± 0.02 | 0.57 ± 0.02 |
| Boosted tree | 0.57 ± 0.02 | 0.58 ± 0.02 |
| Neural network | 0.53 ± 0.02 | 0.58 ± 0.02 |
| Support vector machine | 0.47 ± 0.003 | 0.50 ± 0.003 |

* Refer to Table 1 for the clinical/demographic characteristics that were used for this analysis

**Supplementary Table 4.** Minor allele frequencies of the *SLCO1B1* variant rs4149056 in Singaporean Chinese, Malay and Indian compared to European ethnicities

|  | **Frequency in cases** | **Frequency in controls** |
| --- | --- | --- |
| Singaporean Chinese | 0.0357 | 0.135 |
| Singaporean Malay | 0 | 0.103 |
| Singaporean Indian | 0 | 0.044 |
| European * | 0.52 | 0.13 |

* Data obtained from the seminal GWAS paper ont the association of SLCO1B1 with statin-induced myopathy by Link, et al. (2008)
